# Editorial: Molecular mechanisms of fruit quality formation in fruit trees

**DOI:** 10.3389/fpls.2024.1413866

**Published:** 2024-05-09

**Authors:** Lihu Wang, Lixin Wang, Ze Peng, Xitong Fei, Hairong Wei

**Affiliations:** ^1^ School of Landscape and Ecological Engineering, Hebei University of Engineering, Handan, China; ^2^ College of Horticulture, Hebei Agricultural University, Baoding, Hebei, China; ^3^ College of Horticulture, South China Agricultural University, Guangzhou, China; ^4^ College of Forestry, Northwest Agriculture and Forestry University, Xianyang, China; ^5^ College of Forest Resources and Environmental Science, Michigan Technological University, Houghton, MI, United States

**Keywords:** molecular mechanisms, fruit quality, fruit trees, transcriptomic and metabolomic analysis, fruit development and ripening

Fruits, abundant in various nutrients, play a crucial role in the modern diet and offer numerous health benefits to humans (Yisimayili and Chao, 2022). With the world population booming, there’s a heightened need for fruit quality to meet the escalating global demand. This is because fruit quality largely determines its acceptance and preference among consumers, market share, and economic value. Fruit quality includes both interior and exterior characteristics, including color, sheen, size, texture, flavor, taste, and content of common and/or specific nutrients (Buthelezi et al., 2021; Muhammad et al., 2023). While fruit quality as aforementioned is primarily determined by its genetic composite, it can also be influenced by environmental factors such as temperature, light, humidity, cultivation systems, and certain stresses. As a result, fruit quality is complex trait that has attracted considerable research attention.

Unlike other horticultural crops such as vegetables and flowers, fruit trees generally undergo a lengthy juvenile phase, which present challenges for fruit quality studies. These challenges include, but are not limited to, the extended time required for trees to mature and produce the desired quality of fruit, the complexity involved in conducting experimental designs and assessing fruit quality, and the complications in breeding programs and genetic studies aimed at improving fruit quality. However, with the application and advancement of omics and gene editing technologies, we are unraveling elucidating the molecular mechanisms underlying fruit development and ripening in model plants (Sabbadini et al., 2021; Li et al., 2022; Liu et al., 2022). Yet, there remains a pressing need for a comprehensive understanding of the physio-ecological processes and molecular mechanisms involved in fruit quality formation, especially in fruit trees. Further research advances in this area will greatly facilitate the cultivation of high-quality, nutrient-rich germplasms for developing fruit trees.

The current Research Topic encompassed a total of eight academic articles, focusing on five different fruits: jujube, sour jujube, apricot, loquat, and others. Among these, four articles employed integrated transcriptomic and metabolomic analyses to elucidate the mechanisms associated with fruit quality. One article, compiling and scrutinizing research data from the past decade, revealed that antioxidant activity has the highest co-occurrence with other indicators of fruit quality. Particularly noteworthy is its correlation with anthocyanins, phenolic compounds, sugars, and fruit hardness.

The study conducted by Ni et al. encompassed a comprehensive analysis of fruit quality-related literature spanning from January 2013 to June 2023, employing bibliometric analysis and data mining techniques. The primary objective was to provide guidance for both fundamental and practical research endeavors in the realm of fruit quality. Their research identified tomatoes as the most extensively studied fruit, particularly focusing on topics such as photosynthesis, fruit development, and molecular breeding. The prevailing research direction predominantly centered on exploring the resistance and storage attributes of fruits. A notable finding was the significant role of antioxidant activity as a key determinant of fruit quality, exhibiting strong correlations with other quality-related factors such as anthocyanins, phenolic substances, sugars, and fruit firmness. In another study by Cao et al., the research delved into the biosynthesis of triacylglycerols, revealing insights into the genetic mechanisms. They identified 280 genes encoding seven distinct classes of enzymes involved in this process. showed that 280 genes encoding seven distinct classes of enzymes are involved in the biosynthesis of triacylglycerols. Among them, functional redundancy was observed in some duplicated genes, likely stemming from the large-scale duplication events. Furthermore, the absence of the PDCT pathway in *Vernicia fordii* and *Xanthoceras sorbifolium* was highlighted, setting a groundwork for further studying the functions of the key genes in triacylglycerols-biosynthesis.

The study conducted by Su et al. on anthocyanin and carotenoid accumulation of loquat fruits, which was analyzed through metabolome and transcriptome analyses, revealed that the orange color of ‘Jiefangzhong’ (JFZ) fruit was primarily resulting from carotenoids, while the red color of wild *Eriobotrya henryi* (EH) was attributed to cyanidin-3-O-galactoside and pelargonidin-3-O-galactoside. Furthermore, the variations in transcript levels of *F3H*, *F3’H*, *ANS*, *CHS* and *CHI* contributed significantly to the color distinctions observed between t these fruits. Additionally, two other studies conducted by Chen et al. and Han et al. focused on flavonoid synthesis in apricot fruits across different cultivars, by transcriptome and metabolome analyses. In both studies, many differentially expressed genes (DEGs) and different types of flavonols were identified. Chen et al. highlighted key DEGs related to flavonoids biosynthesis along with identification of three transcription factor genes (2 *bHLHs* and 1 *MYB*) On the other hand, Han et al. identified two transcription factor genes (*PARG27864* and P*ARG10875*) associated with *PARG09190* and *PARG15135*. These findings shed new light on the molecular mechanisms governing flavonoid biosynthesis in apricot fruits, providing valuable insights into this aspect of fruit quality.

The other three studies delved into flavonoid biosynthesis, sugar-acid fractions, and alkaloid metabolism in Chinese jujube fruits. In the study by Muhammad et al., an analysis of the transcription factor MYB family in *Ziziphus. mauritiana* and *Ziziphus jujuba* revealed that the transient expression of *ZjMYB44* in jujube fruits resulted in increased flavonoid content. Yang et al. conducted a study involving a F_1_ population (179 hybrid progeny) of *Ziziphus jujuba* Mill. ‘JMS2’ and (*Z. acido jujuba*) ‘Xing16’, where the analysis of this population identified three additive-dominant major gene and polygenes one associated with glucose synthesis, and two involved in malic acid synthesis. Xue et al. identified 44 differential alkaloid metabolites (DAMs) that were highly and specifically accumulated in sour jujube compared to Chinese jujube. Additionally, transcriptome analysis led to the identification of 259 DEGs, Through the regulatory networks of DAMs and DEGs,11 candidate genes involved alkaloid synthesis and metabolism were identified. These studies collectively offer new insights into the molecular mechanisms shaping jujube fruit quality formation.

In summary, this topic underscores the recent impact of advanced genomic tools and technologies on investigating the mechanisms underlying fruit quality formation. Increased utilization multi-omics approaches and advanced bioinformatics tools for gene family identification advanced our understanding in this fields, offering strong theoretical support for accelerating fruit tree breeding and meeting the rising demand for high-quality fruits.

Collectively, these studies have made significant contribution to the comprehension of secondary metabolite biosynthesis in fruits, laying a solid foundation for further research and potential applications in enhancing fruit quality through genetic improvement and breeding programs.

## Author contributions

LHW: Funding acquisition, Writing – original draft, Writing – review & editing. LXW: Writing – original draft, Writing – review & editing. ZP: Writing – original draft, Writing – review & editing. XF: Writing – original draft, Writing – review & editing. HW: Writing – original draft, Writing – review & editing.
